# Ultrasonographic parameters of the liver, spleen and kidneys among a cohort of school children in Sri Lanka

**DOI:** 10.1186/s12887-017-0943-4

**Published:** 2017-11-16

**Authors:** Dianne Tania Dayanthi Warnakulasuriya, Pattiya Pathirennahalage Uddika Chamila Peries, Yaddehi Arachchillage Chaminda Rathnasekara, KaluArachchige Thushari Madurika Jayawardena, Angappulige Upasena, Anada Rajitha Wickremasinghe

**Affiliations:** 1 Department of Physiology, Faculty of Medicine, University of Kelaniya, Thalagolla Road, Ragama, 10110 Sri Lanka; 2 Department of Radiology, DeSoysa Maternity Hospital, Colombo, Sri Lanka; 3 Rehabilitation Hospital, Ragama, Sri Lanka; 4 Base Hospital, Mirigama, Sri Lanka; 5 Department of Radiology, Colombo North Teaching Hospital, Ragama, Sri Lanka; 6 Department of Public Health, Faculty of Medicine, University of Kelaniya, Thalagolla, Ragama, 11010 Sri Lanka

**Keywords:** Liver, Spleen kidney, Ultrasound parameters, Pediatric imaging

## Abstract

**Background:**

Liver, spleen and kidney dimensions on ultrasonography vary with the age, weight and ethnicity. Reference standards of these parameters for normal Sri Lankan children are not available. Our aim was to establish normative data for longitudinal length of liver, spleen and kidneys in healthy children.

**Method:**

Three hundred fifty-seven children, 5–13 years of age were selected from two randomly selected schools in the Gampaha district in the western province of Sri Lanka. A questionnaire was administered to the parents after obtaining informed written consent. Participants were screened for risk factors for organomegaly and were examined by a trained officer. Children with a past history of infective, inflammatory, haematological, malignant, congestive, collagenous or congenital conditions that can affect the size of the organs were excluded as well as those with clinically evident malnutrition, anemia, lymphadenopathy or organomegaly.

Ultrasonographic assessment was done using a high resolution real-time scanner with a 3.5 MHz convex transducer by a trained officer. Children with ultrasonographic abnormalities of organs were also excluded from the study and referred for further evaluation.

**Results:**

The study comprised 332 children comprising 176 girls (53%). There was a significant difference in the longitudinal dimension of the liver between the two sexes with a higher value recorded among females (Mann Whitney U = 11,830.5, *p* = 0.037). Body weight was correlated with the dimensions of the liver, the spleen and the kidneys. On multiple regression analysis body weight significantly associated with all the organs. (*p* < 0.01) Percentile graphs for longitudinal length of liver, spleen, right and left kidneys were formed according to the body weight.

**Conclusion:**

The organ dimensions showed the highest correlation with body weight. We hope the normal ultrasonographic values of healthy Sri Lankan children will assist in interpretation of sonographic examinations in daily clinical practice.

## Background

The presence of splenomegaly and hepatomegaly is routinely assessed in the pediatric population when there is a suspicion of enlargement of these organs due to infection or malignancy. As bedside methods of palpation and percussion are less reliable [1, 2], they are augmented with ultrasonographic measurements of these organs. The spleen, liver and renal sizes vary by somatic parameters [3, 4]. In the absence of standard measurements of liver, spleen and kidney sizes of Sri Lankan children, interpreting ultrasonographic reports is difficult and may lead to errors in management.

Clinical assessment of the liver is done by palpating the degree of the extension of the liver below the costal margin and the span of dullness on percussion. The normal liver edge can be felt upto 2 cm below the right costal margin and, in a newborn infant upto 3.5 cms below the costal margin in the midclavicular line. The spleen is palpable only when it is two to three times its normal size, although it may be palpable in 10% of healthy children and 15% of neonates [5]. The accuracy of measuring the spleen and liver by palpation and percussion has been shown to be less accurate particularly in detecting small increase in size of the organ [1, 6]. Kidney sizes cannot be measured by examination and only gross enlargement will be detected by ballottement.

Ultrasonography is an easy, inexpensive, noninvasive and accurate method that is commonly used to assess sizes of these intra abdominal organs. The liver is measured in the mid clavicular line with simultaneous demonstration of the right kidney during quiet breathing in young children and during breath holding in older children. The upper and lower points of the sonographic image are taken as the measurement [7]. The spleen is measured in the longitudinal coronal view. The maximal distance between the most supero – medial and infero-lateral points are taken as the spleen length [8]. The lateral decubitus position is preferred to measure the kidney sizes [3, 9].

In Sri Lanka, there are no published data on ultrasonographic measurements of the liver, the spleen and the kidney. Our objective was to describe the ultrasonographic dimensions of the liver, the spleen and the renal length of Sri Lankan school aged children and to determine the relationship between the dimensions of the organs and age, sex and somatic parameters.

## Methods

Three hundred fifty-seven children 5–13 years of age were recruited in to the study from 2 randomly selected schools from the Gampaha district of the western province of Sri Lanka from the list of schools obtained from the zonal educational office in Gampaha. All the children between the ages of 5–13 years studying at the selected schools were recruited in to the study.

A detailed clinical history was taken to exclude the presence of any infective, inflammatory, haematological, malignant, congestive or collagenous conditions that can affect the size of the liver and the spleen. Children with a previous history of acute or chronic hepatitis, jaundice or chronic renal failure were excluded from the study. Children with fever, lymphadenopathy, macular or maculopapular rashes within the past 3 months were also excluded. The imaging criterion for exclusion were abnormalities in the position, shape and echo texture, the presence of parenchymal mass lesions, cysts, accessory spleens, hydronephrosis, or calyectasis.

An interviewer administered questionnaire was used to obtain personal details and past medical history. A trained examiner conducted the physical examination. Weight was measured using a calibrated electronic scale to the nearest 0.1Kg and height was measured using a stadiometer to the nearest 0.1 cm by a trained examiner. Deviation of growth parameters from the normal range demarcated in the child health development record was an exclusion criterion.

Ultrasonographic examination was done using a high resolution real-time scanner (PHILIPS HD 6, Germany) with a 3.5 MHz convex transducer. Liver measurements were performed in a supine position. The longitudinal axis was measured after clear visualization of the liver in the midclavicular plane. The uppermost edge under the dome of the diaphragm was defined as the upper margin, and the lowermost edge defined as the lower margin. Spleen measurements were performed in a lateral decubitus position. The longitudinal measurement of the spleen was taken between the most supero-medial and the most infero-lateral margins. Kidney dimensions were recorded in a lateral decubitus position with the renal hilum visualized to get the optimum longitudinal dimension. The measurements of organ dimensions was made during deep inspiration.Each organ was measured 3 times and the mean value was recorded as the absolute length.

### Data entry and statistical analysis

Data entry was done using Epidata version 3.1 and statistical analysis was done using SPSS/PC version 22.0 (SPSS Inc., Chicago, IL). Descriptive statistics were used to describe the dimensions of organs. The percentiles were determined using frequency. As the age and longitudinal length of the liver were not normally distributed, Mann Whitney U statistics were used for comparison of measurements between the sexes; for comparison of the measurements of the spleen and liver between the sexes, the independent sample t-test were used. The association between organ dimensions and age, weight and height were examined using the Sperarman rank correlation coefficient. Multiple regression analysis using organ dimensions as the dependent variable with age, sex, weight and height as independent variables was carried separately for each organ.

## Results

The parents of all 357 children invited to participate in the study gave consent; 25 children were excluded due to the presence of exclusion criterion (18 due to a past medical history and 7 due to imaging exclusion criterion). Two children with abnormal ultrasonographic appearance of the kidneys were referred to the Paediatric unit of the Colombo North Teaching Hospital. 332 children [176 girls (53%)] between 5 to 13 years (mean [SD] - 7.7[2.0] years) were evaluated. The age and sex distribution of the different weight groups are shown in Table 1.

**Table 1:** Age and sex distribution of the study population by body weight category

Weight group (Kg)	Age (years)				Sex		
	Mean	SD	Median	Inter Quartile (IQ) range	Male	Female	Total
≤20	5.97	1.01	6.00	6.90–7.87	64	62	126
21–30	8.19	1.67	8.00	7.00–9.00	81	77	158
31–40	9.83	1.83	10.00	8.96–11.79	10	31	41
≥41	11.28	1.25	12.00	10.00–12.00	01	06	7
Total	7.66	2.06	7.00	7.30–8.80	156	176	332

The descriptive statistics of the organ dimensions by weight groups are shown in Tables 2, 3, 4, 5. There was no significant difference in longitudinal dimension of the spleen and kidneys between the sexes (*p* > 0.05). The longitudinal length of the liver was significantly higher among the females compared to the males(Mann Whitney U = 11,830.5, *p* = 0.037). The left kidney was 10 mm longer than the right kidney, but the difference was not significant (*p* = 0.073).

**Table 2:** Longitudinal length of the liver by body weight category

Weight group (Kg)	liver length (cm)						95% CI of the mean	
	Median	IQrange	Mean	SD	Min	max	Upper	lower
≤20 *n* = 126	7.40	6.90–7.87	7.44	0.93	5.10	10.4	7.27	7.60
21–30 *n* = 158	8.18	7.64–8.75	8.22	0.91	5.54	10.8	8.07	8.36
31–40 *n* = 41	9.39	8.63–10.20	9.42	1.26	5.42	11.8	9.02	9.81
≥41 *n* = 7	10.00	8.74–10.60	9.85	1.10	8.53	11.5	8.83	10.87

**Table 3:** Longitudinal length of the spleen by body weight category

Weight group (Kg)	Spleen length(cm)						95% CI of the mean	
	Median	IQ range	Mean	SD	Min	Max	Upper	lower
≤20 *n* = 126	6.90	6.38–7.52	6.93	0.84	5.0	9.70	6.78	7.08
21–30 *n* = 158	7.54	7.27–8.13	7.54	0.79	5.41	9.70	7.41	7.66
31–40 *n* = 41	8.55	7.76–9.17	8.46	1.04	5.72	10.7	8.13	8.78
≥41 *n* = 7	9.26	8.64–9.36	9.09	0.52	8.50	9.92	8.61	9.57

Median and Interquartile range(IQR) was provided for clarity and for comparison of results with future studies

**Table 4:** Longitudinal length of the right kidney by body weight category

Weight group (Kg)	Right kidney length(cm)						95% CI of the mean	
	Median	IQ range	Mean	SD	Min	Max	Upper	lower
≤20 *n* = 126	7.12	6.69–7.53	7.12	0.67	5.50	9.36	7.00	7.24
21–30 *n* = 158	7.73	7.27–8.13	7.72	0.58	6.14	9.29	7.63	7.81
31–40 *n* = 41	8.32	8.00–8.69	8.33	0.54	7.05	9.78	8.16	8.49
≥41 *n* = 7	9.14	8.40–9.76	9.10	0.80	8.00	10.30	8.36	9.84

Median and Interquartile range(IQR) was provided for clarity and for comparison of results with future studies

**Table 5:** Longitudinal length of the left kidney by body weight category

Weight group (Kg)	Left kidney length(cm)						95% CI of the mean	
	Median	IQ range	Mean	SD	Min	Max	Upper	lower
≤20 *n* = 126	7.23	6.90–7.59	7.23	0.55	5.10	8.50	7.14	7.33
21–30 *n* = 158	7.87	7.47–8.15	7.81	0.54	6.45	9.07	7.73	7.90
31–40 *n* = 41	8.50	8.01–8.94	8.48	0.64	7.10	9.99	8.27	8.68
≥41 *n* = 7	8.88	8.07–9.40	8.87	0.78	7.81	10.00	8.14	9.59

Median and Interquartile range(IQR) was provided for clarity and for comparison of results with future studies

**Fig. 1:**
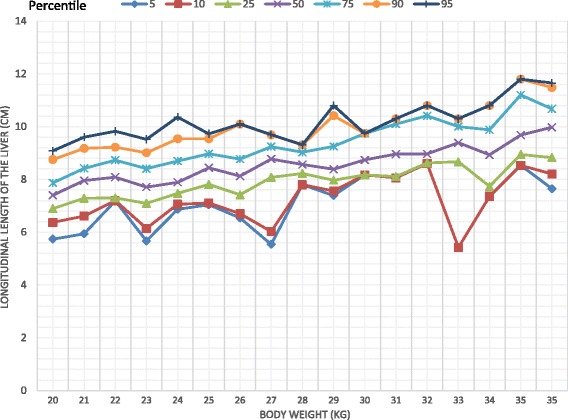
Longitudinal length of the liver by body weight category

**Fig. 2:**
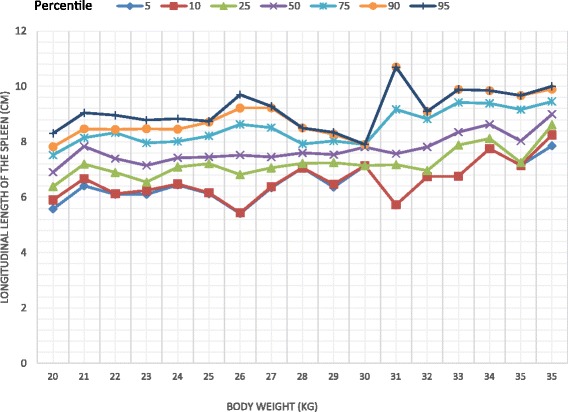
Longitudinal length of the spleen by body weight category

**Fig. 3:**
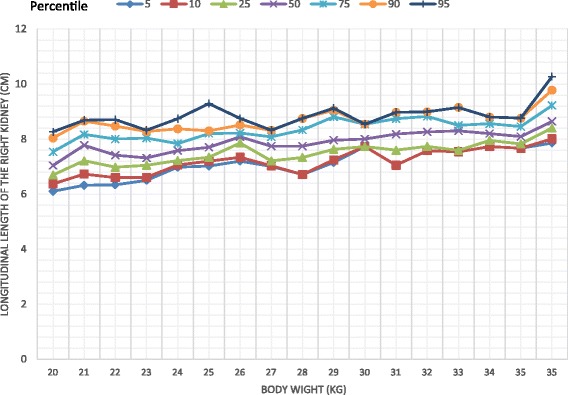
Longitudinal length of the right kidney by body weight category

**Fig. 4:**
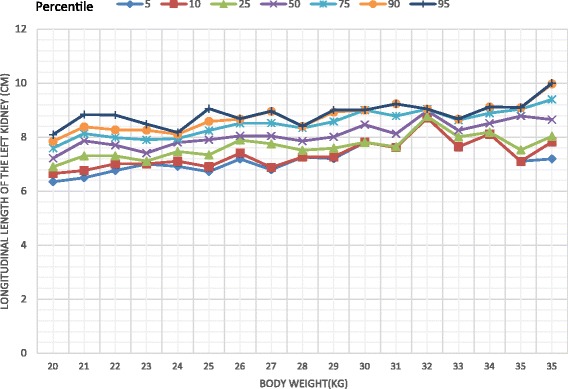
Longitudinal length of the left kidney by body weight category

Body weight significantly correlated with the longitudinal dimensions of the liver (*r* = 0.742, *p* < 0.001), spleen (*r* = 0.604, *p* < 0.001), right kidney (*r* = 0.539, *p* < 0.001) and left kidney (*r* = 0.637, *p* < 0.001). The percentile curves of the liver, spleen and kidneys were defined according to the body weight (Figs. 1, 2, 3, 4). Height was weakly but significantly correlated with liver dimensions (r^2^ = 0.247, *p* < 0.001); BMI was correlated with kidney dimensions (*r* = 0.381, *p* < 0.001 and *r* = 0.403, *p* < 0.001 right and left kidneys respectively). Age was correlated with the longitudinal dimension of the spleen (*r* = 0.359, *p* < 0.001) (Table 6).

**Table 6:** Correlation between organ dimensions and somatic parameters. (Spearman rank correlation coefficient)

		Liver	Spleen	Right Kidney	Left Kidney	Age
Weight	Correlation coefficient	0.579	0.486	0.614	0.624	0.740
	*P*-value	<0.001	<0.001	<0.001	<0.001	<0.001
Height	Correlation coefficient	0.530	0.437	0.591	0.597	0.839
	P-value	<0.001	<0.001	<0.001	<0.001	<0.001
BMI	Correlation coefficient	0.384	0.347	0.350	0.354	0.320
	P-value	<0.001	<0.001	<0.001	<0.001	<0.001
Age	Correlation coefficient	0.527	0.337	0.534	0.538	
	P-value	<0.001	<0.001	<0.001	<0.001

**Table 7:** Multiple regression analysis with organ dimensions as the dependent variable

Organ dimensions (cm)		Regression coefficient	SE	*p* value	95% CI	R^2^
Liver	Constant	6.526	0.807			0.378
	Weight	0.096	0.014	<0.001	0.069–0.123	
	Age	0.142	0.043	0.001	0.058–0.226	
	Height	−0.014	0.009	0.136	−0.032-0.004	
Spleen	Constant	4.034	0.725			0.296
	Weight	0.069	0.012	<0.001	0.045–0.094	
	Age	−0.070	0.038	0.071	−0.145-0.006	
	Height	0.019	0.008	0.024	0.003–0.035	
Right kidney	Constant	4.781	0.505			0.426
	Weight	0.048	0.009	<0.001	0.031–0.065	
	Age	0.045	0.027	0.090	−0.007-0.098	
	Height	0.011	0.006	0.059	0.000–0.022	
Left Kidney	Constant	5.392	0.472			0.416
	Weight	0.050	0.008	<0.001	0.034–0.066	
	Age	0.036	0.025	0.147	−0.013-0.086	
	Height	0.007	0.005	0.200	−0.004-0.018	
Mean Kidney	Constant	5.080	0.424			0.492
	Weight	0.049	0.007	<0.001	0.035–0.063	
	Age	0.041	0.022	0.072	−0.004-0.085	
	Height	0.009	0.005	0.064	−0.001-0.019	

On multiple regression analysis, weight and age were significant predictors of the longitudinal length of the liver after controlling for height. Weight and height were significant predictors of spleen length after controlling for age. In the case of the kidneys, only weight was a significant predictor of length after controlling for height and age, when each kidney was considered separately and when both kidneys were considered together (Table 7).

## Discussion

There are a few studies in the literature that have published normal ultrasonographic parameters of abdominal organs in school aged children but none are available for the Sri Lankan population [4, 10, 11]. Sonography is a common imaging method used in routine practice. The inability to interpret the results due to lack of population norms was a major knowledge gap. Our objective was to define the normal limits of liver, spleen and kidney dimensions in a large group of school aged children. To our knowledge this is the only study of this kind done on Sri Lankan school aged children.

Longitudinal measurements of the liver, spleen and kidneys have been reported to best correlate with body parameters [10, 12–14]. In obtaining measurements of the liver, the longitudinal length at the midclavicular plane has shown the best correlation with body parameters [10, 13, 14] and this was the measurement that we used in this study. Of the different methods for evaluating the kidney by sonography, lateral decubitus position was used preferentially by many previous investigators [9, 10].

Normal percentiles of the liver, spleen and kidneys have been previously described by age [12, 13] and height [4, 14, 15]. We found that the longitudinal parameters of all the organs measured, were highly correlated with body weight, similar to findings of previous studies [14–16]. Age and height were also correlated with organ dimensions but not to the extent of body weight. This is clearly seen in weight being a predictor of the dimensions of all organs, unlike height and age, in the multiple regression analyses which controlled for all the variables. Percentile curves of the liver, spleen and kidneys were thus defined according to body weight categories.

There was no significant difference in the longitudinal measurements of the spleen and kidneys between the sexes as reported by many other authors [10, 14]. However, there was a significant difference in the longitudinal length of the liver between the two sexes. In this study, the longitudinal length of the left kidney was longer than the right, but the difference was not statistically significant; other authors have reported similar findings [10, 11]. Christophe et al. [17] also reported that the difference in the longitudinal length of the kidneys is negligible.

In comparing the normal parameters of abdominal organs on ultrasonography assessment, it was found that the liver length was significantly lower in the present study compared to Dhingra et al. [3] in the weight groups of 10–20 kgs (*p* < 0.001), 20–30 kgs (p < 0.001), 30–40 kgs (p < 0.001), 10–20 kgs (p < 0.001), >40kgs (p < 0.001), The mean spleen length of those in the 30–40 kgs category were significantly different (p < 0.001) to that reported by Dhingra et al., [3], the mean spleen lengths in the other weight groups being similar to Dhingra et al.’s study.

Otive et al. [18] has reported a mean renal length for children in India by age. The mean renal length in the present study by age was compared with findings reported by Otive; there were significant differences in the 5 year (*p* = 0.048), 6 and 7 year (p < 0.001 in both) age groups but not in the age groups from 8 to 12 years. This confirms that even data on ultrasonographic assessment of abdominal organs reported from South Asian countries differ. Ideally, establishing normograms for each ethnic group will enable better interpretation of sonographic assessments in the pediatric population.

## Conclusion

Longitudinal parameters of liver, spleen and kidneys correlated with body weight even after adjusting for height and age. Age and height were also correlated with organ dimensions. There was a statistically significant difference of the longitudinal length of the liver between the two sexes. The difference in the longitudinal length of the kidneys was negligible.

The results of this study may be used as a guide to interpret the normal sizes of the liver, the spleen and the kidneys of school aged Sri Lankan Sinhalese children based on body weight categoriess. As the study population comprised only Sri Lankan Sinhalese children residing in the Gampaha district, an island wide survey including all ethnicities needs to be conducted to generate percentile graphs for generalized use.

## Abbreviations

BMI: Body Mass Index
CI: Confidence interval
SD: Standard Deviation
SE: Standard error
